# Carotid plaque inflammatory activity assessed by 2-[18F]FDG-PET imaging decrease after a neurological thromboembolic event

**DOI:** 10.1186/s13550-021-00773-y

**Published:** 2021-03-23

**Authors:** Laerke Urbak, Rasmus S. Ripa, Benjamin V. Sandholt, Andreas Kjaer, Henrik Sillesen, Martin Graebe

**Affiliations:** 1 Department of Vascular Surgery, Copenhagen University Hospital, Blegdamsvej 9, 2100 Copenhagen, Denmark; 2 Department of Clinical Physiology, Nuclear Medicine and PET and Cluster for Molecular Imaging, Rigshospitalet and University of Copenhagen, Copenhagen, Denmark

**Keywords:** FDG PET, 3D ultrasound, Carotid artery plaque, Inflammation, Vulnerable plaque

## Abstract

**Background:**

Atherosclerotic plaque vulnerability is comprised by plaque composition driven by inflammatory activity and these features can be depicted with 3D ultrasound and 2-[18F]FDG-PET, respectively. The study investigated timely changes in carotid artery plaque inflammation and morphology after a thromboembolic event with PET/CT and novel ultrasound volumetric grayscale median (GSM) readings. Patients with a single hemisphere-specific neurological symptom and the presence of an ipsilateral carotid artery atherosclerotic plaque were prospectively included to both 2-[18F]FDG PET/CT and 3D ultrasound scans of the plaque immediately after their event and again three months later. On PET/CT images the maximum standardized uptake value (SUV_max_) was measured and the volumetric ultrasound acquisitions were analyzed using a semiautomated software measuring GSM values.

**Results:**

Baseline scans were performed by a mean of 7 days (range 2–14) after the symptom and again after 98 days (range 91–176). For the entire group (n = 14), we found a decrease in average SUV_max_ from baseline to follow-up of − 0.18 (95% confidence interval: − 0.34 to − 0.02, *P* = 0.034). GSM did not increase significantly over time (mean change: + 2.21, 95% confidence interval: − 17.02 to 21.44, *P* = 0.808).

**Conclusion:**

A decrease in culprit lesion 2-[18F]FDG-uptake 3 months after an event indicates a decrease in inflammatory activity, suggesting that carotid plaque stabilization over time. 3D ultrasound morphological quantitative differences in GSM were not detectable after 3 months.

## Introduction

Symptoms as transient ischemic attack, minor ischemic stroke or amaurosis fugax will in some patients precede a major disabling stroke [1, 2]. Therefore, when a concomitant stenotic plaque in the carotid artery ipsilateral to the symptomatic hemisphere is believed to be the culprit lesion, immediate prophylactic measures are indicated. Depending on degree of stenosis, symptoms and demographic data, patients are either treated with carotid endarterectomy (CEA), carotid artery stenting (CAS) or in most cases by medical treatment only [3]. Most thromboembolic events arise from highly inflammatory active atherosclerotic lesions regardless of size or degree of stenosis [4, 5]. Among other inflammatory cells macrophages have shown to play a central role in plaque destabilization causing degeneration of the fibrous cap and expansion of the necrotic core [6, 7] that may lead to plaque rupture or erosion [8]. Thus, atherosclerosis, and in particular symptomatic atherosclerosis, may be considered an inflammatory disease [9]. Sub-analyses of large randomized trials have shown that CEA is most beneficial within the first weeks after symptom onset [10], indicating that the inflammatory activity leading to plaque vulnerability is a dynamic process that may diminish or increase rapidly over time. It is not known which factors influence inflammatory activity, although recent years improvement in medical prophylactic treatment is believed to have an impact on both plaque stabilization and inflammatory activity.

Molecular imaging with positron emission tomography (PET) or structural imaging with ultrasound (US) is capable of identifying inflammation and other features of plaque vulnerability. PET in conjunction with computed tomography (CT) is used in daily clinical practice to depict specific cellular metabolism on a molecular level. The tracer [^18^F]-fluoro-2-deoxyglucose (2-[18F]FDG) is a glucose analogue that accumulates in high-glucose-utilizing cells and we have previously shown that 2-[18F]FDG-uptake in symptomatic carotid plaques is correlated to inflammatory activity and macrophage abundance [11–13]. Statin treatment has shown to decrease the 2-[18F]FDG-uptake in carotid plaque over 3–6 months [14, 15]. Furthermore, 2-[18F]FDG-uptake is increased in symptomatic carotid plaques compared to contralateral asymptomatic plaques [16, 17], suggesting that PET/CT 2-[18F]FDG-uptake quantification is a highly specific marker of plaque vulnerability and inflammation.

Ultrasound imaging can detect morphologic features of carotid plaques with increased vulnerability and high inflammatory activity. With conventional two-dimensional (2D) US, low echogenicity has been found in symptomatic carotid plaques [18–20] and quantification of echogenicity can be used to calculate future stroke risk [21, 22]. To quantify the echogenicity of plaques, grayscale median (GSM) measurements is commonly used: A high GSM measurement (compatible with high echogenicity) indicates a large amount of fibrotic and calcified tissue [23] as found in “silent” or asymptomatic plaques, whereas low echogenicity and GSM corresponds to a high content of soft atheroma and lipids [20, 24, 25] which are typical features of the symptomatic or vulnerable plaque. In 2D US only a single image is captured for GSM analyses, which is a hindrance for reproducibility and sensitivity in assessing risk on an individual patient-specific level. In the current study, we implemented a novel three-dimensional (3D) US volumetric GSM evaluation of the plaques to improve sensitivity and reproducibility [26, 27].

In the present study we hypothesized that inflammatory activity, in suspected culprit lesions depicted with PET, will diminish over a 3-month period of time on medical treatment following an acute neurological event. Furthermore, it was hypothesized that as inflammatory activity decreases, the plaque will stabilize leading to an increase in the amount fibrous tissue and thereby an increase in volumetric GSM in 3D ultrasound acquisitions.

## Materials and methods

### Patients

We prospectively included patients referred to the Department of Vascular Surgery (Copenhagen University Hospital, Copenhagen, Denmark) from regional neurological departments. Patients were referred in accordance with the regions fast-track protocol for diagnosis and treatment of symptoms of cerebral ischemia caused by ipsilateral atherosclerotic carotid plaques. Patients eligible to study inclusion were those with recent symptoms of minor ischemic stroke, transitory ischemic attack or amaurosis fugax and an ipsilateral carotid plaque defined as any atherosclerotic lesions independent of degree of stenosis. Only patients who were initially deemed not suitable for CEA were included, and in order to improve PET scans, exclusion criteria were dysregulated diabetes (HbA1c > 13.3 µmol/L), blood glucose > 11 mmol/L, infection, cancer or vasculitis, renal insufficiency (creatinine > 125 µmol/L) or known allergy to the used CT-contrast.

The 2-[18F]FDG PET/CT and 3D US scans were performed ≤ 14 days after the last neurological symptom and scheduled again 3 months later. The two scans were performed the same day. Patients files were examined for new cardiovascular events 6 months after the last follow-up scan. We selected a 14-day limit for the first scan as this is the time where CEA is most beneficial [10] suggesting changes in plaque morphology already after the two weeks. All patients immediately started medical treatment with statin and anti-platelet medication after initial hospital admission to neurological departments. Medical history, current symptoms, previous known atherosclerotic risk factors and medical use were obtained for all patients. Hypertension, diabetes, previous heart disease and stroke were defined present if noted in the patient’s file. Smoking history and cessation was registered according to patients’ intimation. Degree of carotid stenosis was measured with duplex ultrasound using peak systolic velocity and end diastolic velocity in accordance with local and common Doppler ultrasound criteria [3]. Only the symptomatic carotid artery was scanned; thus, no contralateral plaques were included (if present).

### Ultrasound

#### Image acquisition

Patients were scanned in supine position with the head turned slightly to the contralateral side. The transducer was a novel (at the time being under development and not yet commercially available) vascular matrix transducer (XL14-3 xMATRIX array transducer, Philips Healthcare, Bothell, WA, USA) supporting 3D imaging by scanning in two directions at the same time creating a block of the artery without any moving parts. The entire extracranial carotid artery was scanned, and the 3D image acquisitions were centered on the largest plaque. The images were stored for later analysis. All US acquisitions were performed by the same investigator (LU). The transducer was tested clinically with authorization from the Danish Health and Medicines Authority.

#### Image analysis

The US image analyses were carried out by the same author who also performed the US acquisitions (LU). To increase reliability and blind data co-author BS manually anonymized US images for patient data and the order of the scans before the image analyses. An offline research software, developed by Philips Research France, Medisys (Suresness, France), was used for image analysis. The common carotid artery (CCA), the flow divider of the bifurcation, proximal and distal internal carotid artery (ICA) and the proximal and distal part of external carotid artery (ECA) were marked by the user. The vessel wall was automatically outlined, visually inspected and adjusted manually. Plaque was then outlined, inspected and adjusted and plaque in ECA was excluded. The plaque volume was segmented axially in 2-mm blocks according to the flow divider permitting data to be aligned with data from the PET/CT image.

**Fig. 1:**
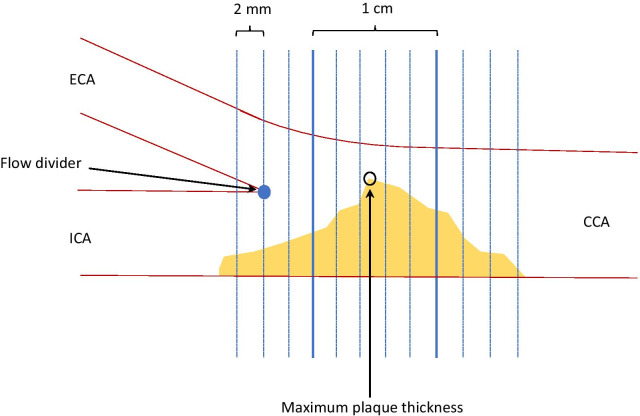
Schematic example of the calculated average max standardized uptake value (aSUV_max_) and grayscale median (GSM). ECA = external carotid artery. ICA = internal carotid artery. CCA = common carotid artery. The 3D ultrasound acquisition of the plaque was sliced in 2-mm segments from the flow divider, and the slice containing the maximum plaque thickness was identified. The slice with the maximum plaque thickness and the two slices on each side were used in analyses. For the PET/CT, the flow divider was used as landmark for alignment of SUV_max_ readings to the ultrasound readings. An average SUV_max_ was calculated for this 1 cm long plaque volume. GSM was automatically calculated by the software for the volume of the 10-mm-long plaque segment

The software automatically provided measurements for the segmented blocks: Volume, maximum plaque thickness and GSM. As each ultrasound scan was individually adjusted before acquisition to optimize quality of brightness, a normalization of the gray scale was performed to secure a standardized comparability of GSM readings between patients. The normalized GSM values were computed by scaling the original GSM value using a factor 190/*X*, where *X* denote the average gray value (0–255) of the adventitia. The maximum plaque thickness was detected, and the software automatically calculated the normalized GSM for the 1 cm volume around the maximum plaque thickness (Fig. 1). Partial plaque volume sampling has previously, in a similar setting, been found to increase reproducibility of 3D volumetric plaque analyses [28].

### PET/CT

#### Image acquisition

After 6 h fasting, 4 MBq/kg 2-[18F]FDG was injected intravenously. To reduce tracer uptake in the muscles near neck and jaw, patients rested without talking for 30 min after injection. Two hours after 2-[18F]FDG administration patients were placed in a combined PET/CT scanner (Siemens Biograph mCT, Siemens, Erlangen, Germany) in supine position with the arms down the side and head fixed. First, an attenuation correction CT scan (120 keV, 50mAs) with 2-mm slices was performed. Then PET bed position of 5 min over 15 cm was performed, and for perfect postprocessing fusion and visualization of the carotid artery high resolution, CT scan (120 keV, 200mAs) in arterial phase with contrast (100 ml Optiray [patients 70–100 kg: 300 mg iodine/ml and patients > 100 kg: 350 mg iodine/ml]; Guerbet, F-9342 Villepinte, France) was performed over the neck.

#### Image analysis

PET/CT images were analyzed by a trained investigator (RSR) blinded to patient data and scan time. After automatic fusion of PET and CT images, OSIRIX MD (Pixmeo SARL, Bernex, Switzerland) regions of interest (ROIs) were manually placed on each 2-mm slice around the CCA and ICA. The first slice where the ECA and ICA separated was noted as the flow divider used for US alignment. For 2-[18F]FDG-uptake, the maximum standardized uptake value (SUV_max_) was computed for each ROI. Images were not corrected for partial volume effect.

The 2-mm-thick volume plaque slices from the ultrasound dataset were manually aligned with the PET slices according to the flow divider. An average SUV_max_ (aSUV_max_) was calculated for the slice with the maximum plaque thickness and the two slices adjacent, on each side (Fig. 1).

### Statistics

Image data (aSUV_max_ and GSM) were tested for normal distribution with histograms and residual plots and presented as mean with standard deviation (SD). Differences in aSUV_max_ and GSM were investigated in a linear mixed model with time as fixed effect and with an unstructured covariance to account for the correlation between replicated measurements on the same subject. This is presented with mean change and 95% confidence interval (CI 95%). Analysis of covariance (ANCOVA) was used for post hoc analysis to estimate whether population means of the dependent variables (aSUV_max_ and GSM) were the same across levels of independent variables (time), adjusting for difference in co-variates as sex, age, body mass index (BMI), smoking, hypertension, statin treatment and degree of carotid stenosis. To test for linear correlation between aSUV_max_ and GSM a linear regression model was used for baseline data, follow-up data and delta data (i.e., change in dependent variables over time). Significance level was set at 0.05. Statistical analyses were performed using SAS enterprise guide 7.1 (SAS institute, USA).

## Results

**Fig. 2:**
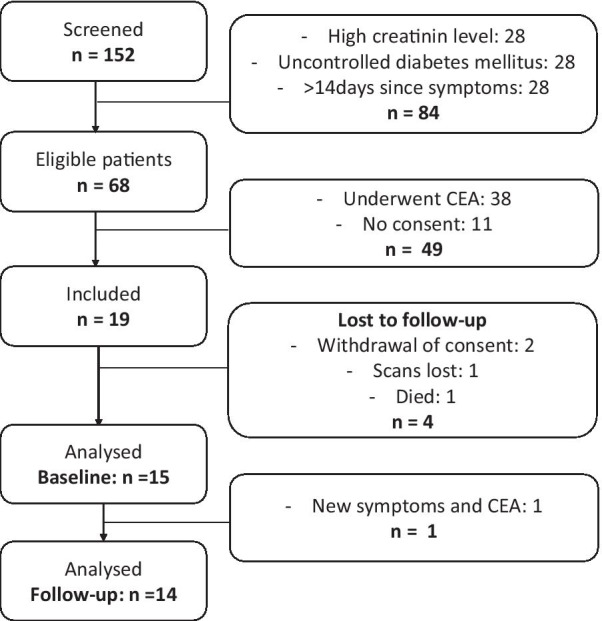
Flowchart of the included patients. Of the 19 included four were excluded. Two were excluded after withdrawal of consent, one died because of an, at the time of inclusion, undiagnosed cancer and one scan was lost

In a two-year period between 2016 and 2019, we included 19 symptomatic patients whereof 15 were analyzed at baseline (Fig. 2) and 14 again at follow-up. All included patients were referred from a neurological department, diagnosed with stroke (53%) or transitory ischemic attack (TIA) (47%), and all were started in medical treatment with statin and antiplatelet therapy before referral to dept of vascular surgery. In 8 patients, CEA was not indicated due to a low degree of stenosis < 50%. For the remaining 7 patients, the vascular surgeon refrained from CEA because of a moderate degree of (near 50%) stenosis and type of symptoms, taken together with the patients general condition. Patients included (Table 1) were primarily male sex and were scanned by a mean of 7 days (range 2–14) after symptom onset and again after a mean of 98 days (range 91–176).

**Table 1:** Demographic data

	*N* = 15 patients
Male sex	14 (93%)
Age (years)	71.2 (54–87)
BMI	27.43 (18.65–36.29)
Smoking	
Currently	2 (14%)
Ever	12 (79%)
Diabetes mellitus	1 (1%)
Hypertension	11 (73%)
History of coronary disease	2 (13%)
COPD	1 (1%)
Previous TIA	1 (1%)
Previous stroke	1 (1%)
Newly diagnosed atrial fibrillation	3 (20%)
Statin lowering treatment*	
Low intensity	8 (53%)
High intensity	7 (47%)
Statin treatment before current stroke/TIA	6 (40%)
Total cholesterol (mmol/L)	4.78 (2.4–6.7)
LDL cholesterol (mmol/L)	2.72 (0.8–3.5)
HDL cholesterol (mmol/L)	1.50 (0.61–4.7)
Creatinine (µmol/L)	88.00 (55–116)
GFR (mL/min)	72.62 (50–93)
Leucocytes (/L)	7.86 (4.3–10.9)
CRP (mg/L)	5.81 (1–16)
Carotid artery with stenosis degree > 50%	7 (46%)

Data are given in mean and range or percentage in brackets

BMI = body mass index, COPD = chronic obstructive lung disease, TIA = transitory ischemic attack

^*^Treatment after neurological symptoms. Low intensity defined as Simvastatin ≤ 40 mg or Atorvastatin ≤ 20 mg. High intensity was defined as Simvastatin > 40 mg and Atorvastatin > 20 mg

Blood samples are missing for two patients

Carotid stenosis degree > 50% was defined as a peak systolic velocity > 125 cm/second

**Fig. 3:**
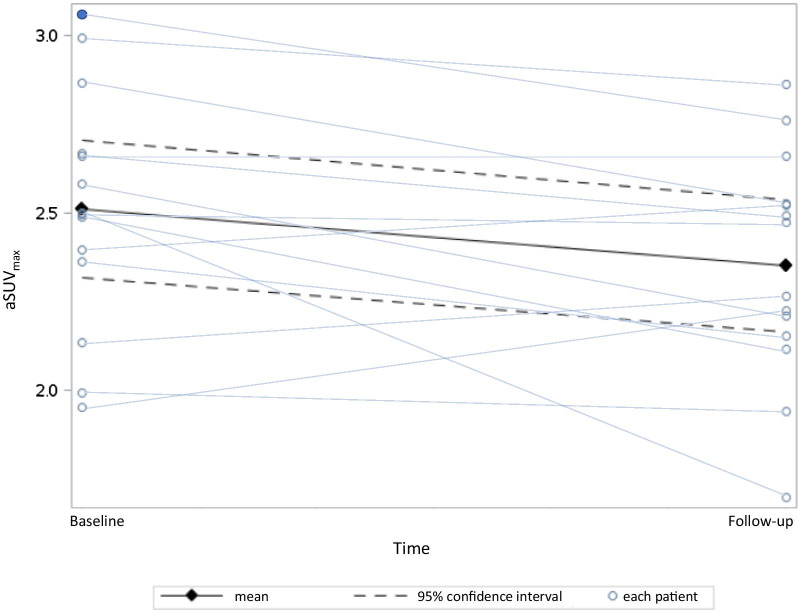
Change in average maximum standardized uptake values (aSUV_max_) from baseline to follow-up. The black diamonds represent the mean aSUV_max_ at baseline and follow-up with the 95% confidence interval as the dotted line. Mean difference in aSUV_max_ of − 0.18, CI95%: − 0.34 to − 0.02, *P* = 0.034. The light blue lines represent each patient with the circles marking the aSUV_max_ at baseline and follow-up. The patient with recurrent symptoms undergoing carotid endarterectomy before the follow-up scan is marked as a filled circle

**Fig. 4:**
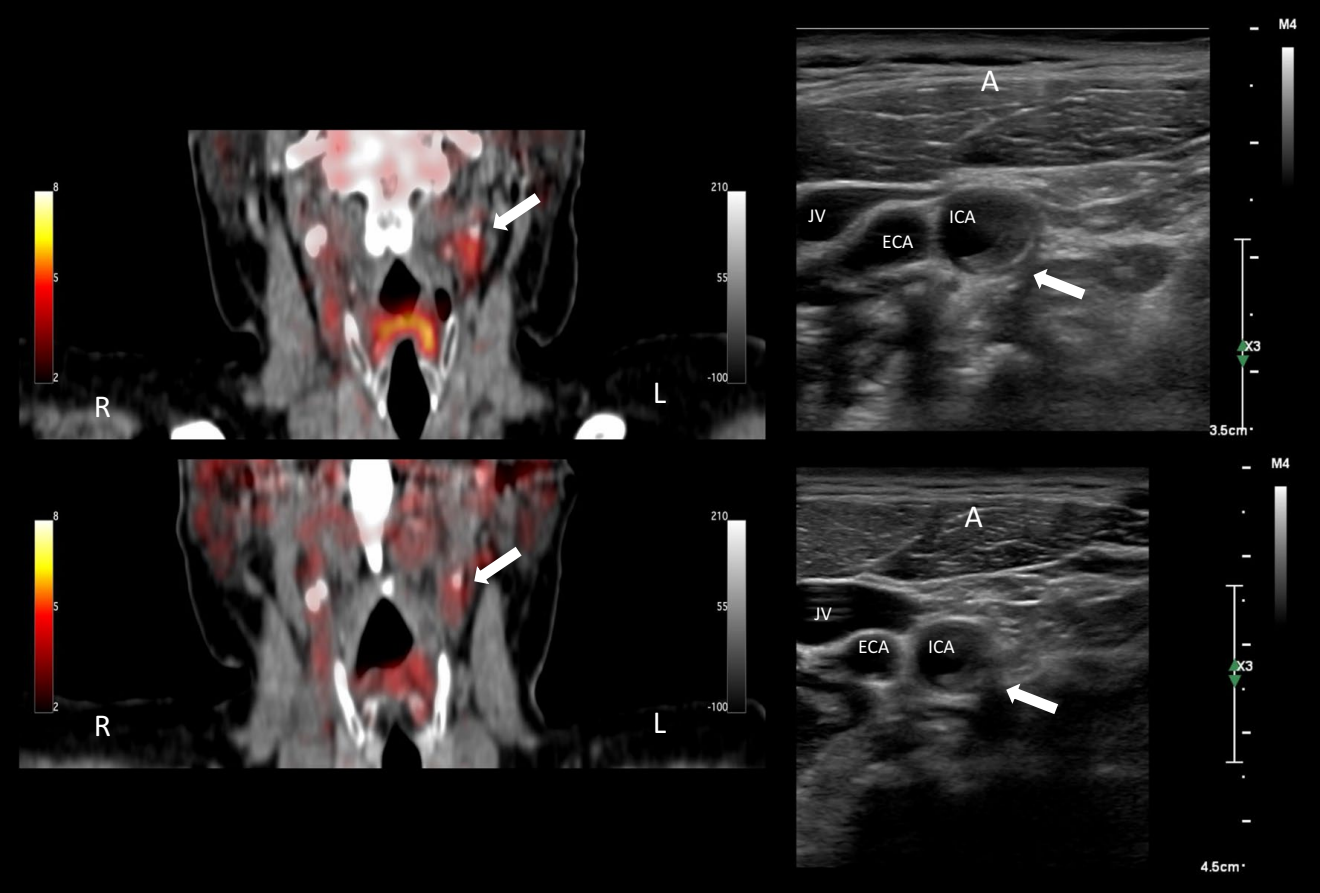
Baseline and follow-up patient example of 2-[18F]FDG-PET / CT-angiography and ultrasound images. Images from one patient. To the left 2-[18F]FDG-PET/CT angiography fusion, frontal MPR and to the right ultrasound images, axial plane. The top images are from baseline and the bottom follow-up. The white arrow points to the plaque on the symptomatic side

For the entire patient group, we found a decrease in aSUV_max_ from baseline (mean: 2.56, SD: 0.37) to follow-up (mean: 2.35, SD: 0.32) (mean change: − 0.18, CI 95%: − 0.34 to − 0.02, *P* = 0.034) on the symptomatic artery (Fig. 3). No change of 2-[18F]FDG-uptake (mean change: − 0.05, CI 95%: − 0.28 to 0.17, *P* = 0.613) was found analyzing the 12 contralateral asymptomatic arteries (two patients had occluded carotid and one no plaque). GSM did not increase significantly from baseline (mean: 64.60, SD: 23.56) to follow-up (mean: 65.86, SD: 36.35) (mean change: 2.21, CI 95%: − 17.02 to 21.44, *P* = 0.808). In a post hoc analysis neither male sex, age, body mass index (BMI), smoking, hypertension, high-dose statin treatment, statin treatment before neurological symptom nor degree of stenosis showed an impact on either outcome. Neither did time in days between scans have an impact on outcome. An example of both image modalities is shown in Fig. 4.

There was no correlation between aSUV_max_ and GSM at baseline (*P* = 0.414), follow-up (*P* = 0.308) nor when comparing the change in aSUV_max_ and GSM over the period of time (*P* = 0.291).

One included patient experienced repeated mild neurological symptom after baseline scanning and was after re-evaluation treated with CEA before the second scan. This patient had a higher aSUV_max_ at baseline (3.19) than any other patient included. GSM was 84 in this patient. None of the 14 patients with both scans had recurrent symptoms in the time between the primary scan and the follow-up scan, nor any new symptoms in a 6-month period after the follow-up scan.

## Discussion

This study shows that 2-[18F]FDG-uptake in symptomatic carotid plaques decreases during a 3-month period after initiation of non-surgical prophylactic treatment. The decrease in inflammatory activity did not lead to a detectable change in plaque morphology quantitated by novel 3D ultrasound plaque GSM measurements.

The study was scheduled to 2 years inclusion and due to low inclusion rate, it did not meet pre-calculated power (40 participants) to detect a minimal relevant difference in GSM measurements. Experience from the present and previous studies of PET scans in patients with a recent minor stroke is that the scan is cumbersome and time-consuming from the often-fragile patients’ point of view. Thus, population selection in conjunction with the conservative inclusion and exclusion criteria explains the poor inclusion rate. Despite being underpowered, it is the authors opinion from data acquisitions and image analyses that 3D GSM measurements in our current setting is not sensitive enough as a standalone to detect significant changes in plaque morphology. Also, the relatively short time frame of 90 days set in the present study might have been too short for detecting plaque substance changes. Previous morphologic plaque studies using MRI have shown carotid plaque lipid-rich necrotic cores are substantially reduced after 12-month medical treatment, even though plaque volume is not reduced [29]. We are currently developing supplemental techniques to investigate other morphological features pivotal for identification of the vulnerable plaque. Volumetric assessment of plaque surface and ulcerations would improve sensitivity further, as would ultrasound contrast enhanced acquisitions in order to accurately display fibrous cap thickness and echo-poor plaque areas that otherwise might be missed because of resemblance to blood echogenicity as both are black in B-mode imaging.

The association between high 2-[18F]FDG uptake in carotid artery plaques, a high inflammatory activity and vulnerability leading to thromboembolic events has been established from studies of molecular pathology to recent clinical studies [11, 16, 17, 30]. In previous studies a change in 2-[18F]FDG uptake of around 10% has been observed with statins and pioglitazone [31–33], drugs that have a proven effect on cardiovascular endpoints. This indicate that a change of around 10% in 2-[18F]FDG uptake could potentially have clinical relevance. Use of upcoming more specific inflammatory tracer might have been able to detect a greater change over time [34–36] which will be of interest in future studies. The use of the maximal standardized uptake value (SUVmax), which takes patient weight and isotope decay into account, is a well-established marker of activity. It can be discussed if, e.g., target-to-background ratios is superior as it takes the background uptake in consideration. However, background activity in the blood pool differ grossly between subjects and clearance of 2-[18F]FDG differs with individual blood glucose level, current level of liver metabolism, etc., and is therefore not ideal either. One measure has not been found superior to the other. When using SUVmax the tomography should, as done in this study, be delayed to a minimum of 2 hours after injection of the isotope to diminish any *spill-over* activity from adjacent tissue and the blood pool [37].

An additional finding was that the one patient with recurrence of neurological symptoms, who was operated with thromboendarterectomy during the follow-up period, had the highest baseline 2-[18F]FDG-uptake. This single finding corroborates the results of the larger study by Kelly et al. suggesting the feasibility in using plaque 2-[18F]FDG-uptake quantification for qualitative individual risk assessment [30]. Seemingly, the method of 2-[18F]FDG PET in carotid plaques in symptomatic patients shows high sensitivity in detecting vulnerable plaques and patients at risk for recurrence. Specificity, on the other hand is questionable, especially in the present study, with no ascertainment other than clinical presentation, that the suspected carotid plaque actually is the culprit lesion and two patients having newly diagnosed atrial fibrillation. Another limitation with 2-[18F]FDG PET is the radiation dose of 6–8 mSv. This will most likely decrease with newer more sensitive tracers and the higher age of relevant patients will in most cases make the radiation less problematic.

The fluctuating changes in inflammatory activity in atherosclerotic disease are yet not understood, and the molecular pathology behind the diminished activity that occurs in some patients as plaques stabilizes and no further events are experienced, are not fully enlightened. Clinical data points toward a pleiotropic (possibly anti-inflammatory) effect of the HMG-CoA reductase inhibitors [38] and statins have shown to decrease 2-[18F]FDG-uptake in plaques in asymptomatic patients [14, 15]. Therefore, although we studied the culprit lesion in symptomatic patients, it is plausible to presume that the effect of immediate prophylactic medical treatment in patients with symptomatic atherosclerosis is, to some extent, derived from statins. However, in our population, six patients were already on statin treatment before their primary neurological event, underscoring the multifactorial influence on atherosclerotic plaque disease progression and regression. Underlining what statin treatment does not eliminate cardiovascular events and suggest that the change in 2-[18F]FDG-uptake in part are due to the natural history of a culprit lesion which have not been shown before.

## Conclusion

2-[18F]FDG-uptake decreases in suspected carotid artery atherosclerotic culprit lesions 3 months after symptoms. The decrease in inflammatory activity and thereby suggested plaque stabilization over time when studying only one aspect of the vulnerable plaque was not detectable in plaque morphology using ultrasound volumetric GSM readings.

## Data Availability

The data supporting the findings of this study are not publicly available as it may contain personally identifiable information.

## Abbreviations

2-[18F]FDG: [18F]-fluorodeoxyglucose
2D: Two-dimensional
3D: Three-dimensional
aSUVmax: Average maximum standardized uptake value
CCA: Common carotid artery
CEA: Carotid endarterectomy
CT: Computed tomography
ECA: External carotid artery
GSM: Grayscale median
ICA: Internal carotid artery
PET: Position emission tomography
US: Ultrasound
